# Multi-dimensionality of chronic pain of the oral cavity and face

**DOI:** 10.1186/1129-2377-14-37

**Published:** 2013-04-25

**Authors:** Joanna M Zakrzewska

**Affiliations:** 1 Facial pain unit, Division of Diagnostic, Surgical and Medical Sciences, Eastman Dental Hospital, UCLH NHS Foundation Trust, 256 Gray’s Inn Road, London, WC1X 8LD, UK

**Keywords:** Facial pain, Temporomandibular disorders, Trigeminal neuralgia, Burning mouth syndrome, Neuropathic pain, Persistent idiopathic facial pain, Cognitive behaviour therapy, Biopsychosocial

## Abstract

Orofacial pain in its broadest definition can affect up to 7% of the population. Its diagnosis and initial management falls between dentists and doctors and in the secondary care sector among pain physicians, headache neurologists and oral physicians. Chronic facial pain is a long term condition and like all other chronic pain is associated with numerous co-morbidities and treatment outcomes are often related to the presenting co-morbidities such as depression, anxiety, catastrophising and presence of other chronic pain which must be addressed as part of management . The majority of orofacial pain is continuous so a history of episodic pain narrows down the differentials. There are specific oral conditions that rarely present extra orally such as atypical odontalgia and burning mouth syndrome whereas others will present in both areas. Musculoskeletal pain related to the muscles of mastication is very common and may also be associated with disc problems. Trigeminal neuralgia and the rarer glossopharyngeal neuralgia are specific diagnosis with defined care pathways. Other trigeminal neuropathic pain which can be associated with neuropathy is caused most frequently by trauma but secondary causes such as malignancy, infection and auto-immune causes need to be considered. Management is along the lines of other neuropathic pain using accepted pharmacotherapy with psychological support. If no other diagnostic criteria are fulfilled than a diagnosis of chronic or persistent idiopathic facial pain is made and often a combination of antidepressants and cognitive behaviour therapy is effective. Facial pain patients should be managed by a multidisciplinary team.

## Introduction

This review will look at pain that predominantly presents in the lower part of the face and the mouth. The epidemiology and classification will be discussed and the diagnostic criteria presented together with a brief mention of management. The review will include a discussion about the multidimensionality of facial pain as there is increasing evidence throughout the field of chronic pain that psychosocial factors impact significantly not just on outcomes from management but also act as prognosticators and can even affect the way symptoms are reported. Many patients will have more than one pain diagnosis and there may also be an underlying psychiatric or personality disorder which pre disposes to chronic pain and which will alter the presentation and significantly affect management [1].

When problems arise in this area patients become very confused as they are unsure as to whether they should consult a doctor or dentist. Equally health care professionals often struggle as it is rare for medical students to be taught in depth about the mouth and surrounding structures. On the other hand dentists do not have in depth knowledge of the biopsychosocial approach to head and neck pain, remain confused about management of non-dental pain and are very restricted in the types of drugs that they can prescribe [2,3]. Hence as Hals et al. [4] point out these patients often get stigmatized as “difficult” as few health care professionals feel capable of helping them single handed as they really need a multi-professional team. A recent study of the healthcare “journey” of chronic orofacial pain patients in the UK showed that 101 patients had attended a mean of seven health care settings, seen a mean of three specialists and only 24% judged their treatment to be successful [5]. This is supported by a similar survey of German University dental schools in 2004 (poor response rate 45% of schools and based on 34,242 patients) that showed that patients were inadequately treated prior to referral but also indicated that many once in the dental schools were still not managed according to guidelines. Most were managed using only one modality despite 30% having psychological morbidity. Only 11% were referred for psychiatric or psychotherapy, 9% for pain therapy and 7% for neurological assessment whereas 30% were referred to oral and maxillofacial surgeons [6].

## Review

### Epidemiology

Aggarwal et al. [7] in a population based study in the UK using a postal questionnaire looked at frequently unexplained pains and found 7% of patients reported having a chronic orofacial pain, 15% reported chronic widespread pain, 9% irritable bowel syndrome and 8% chronic fatigue. Of these 9% reported more than one of these pains. A recent German population study using strictly defined criteria and face to face interviews by trained headache neurologists suggests that trigeminal neuralgia is commoner than persistent idiopathic facial pain but both are rare with a lifetime prevalence of trigeminal neuralgia of 0.3% versus 0.03% for persistent idiopathic facial pain [8]. Koopman et al. [9] using the research databases of all primary care physicians in the Netherlands searched for all cases of facial neuralgias and persistent idiopathic facial pain and found an incidence rate of 38.7 per 100,000 people years The diagnosis were validated by pain experts as they found up to 48% had been misdiagnosed by the primary care physicians. Trigeminal neuralgia and cluster headache were the most common types. On the other hand temporomandibular pain and muscle disorder-type pain (TMD) is common and population-based studies among adults report that approximately 10–15% have these disorders [10,11].

### The pain “journey” and co-morbidity in chronic orofacial pain patients

It is crucial to remember that every long term condition including pain results in psychological morbidity and reduced quality of life. Increasingly it is being recognised that some patients have an increased risk of developing chronic facial pain [12] and recognising this group early may reduce multiple referrals and inappropriate management. It is known that predictors of poor outcome can be identified early [13,14]. A chronic pain patient who finally reaches a multi-disciplinary pain clinic will often have a long duration of pain, with significant functional impairment and will have developed fixed ideas on cause, location and legitimacy of the pain and this will impact on the pain specialists approach [15]. Many as a result will have low expectations from their pain consultation [16]. Studies of chronic facial pain patients have shown similar breakdown in doctor patient relationships and patients express confusion about the varied views they had received on management of their pain and likely outcomes [17]. There is a general desire by orofacial pain patients to be understood, their pain to be acknowledged as real and to feel cared for as life has become hopeless and trust in the medical profession has been lost [18]. Patients expectations for outcomes from a pain clinic will vary and these need to be recognised so management plans are appropriate [19]. Illness beliefs have been found to affect outcomes in patients with orofacial pain [20].

Mental health status will affect the pain experience and conditions that are especially significant are depression and anxiety [21]. It is now known that there are neural markers for fear and anxiety which exacerbate chronic pain [22]. Mental defeat (a psychological construct which includes catastrophising) increases distress and disability from pain [23,24]. Patients with borderline personalities report higher pain levels than other pain patients [25]. A recent study by Taiminen et al. [1] of 63 patients with burning mouth syndrome or atypical facial pain supported these findings. They showed that over 50% of these patients had a lifetime mental health disorder especially depression and personality disorders were common. They demonstrated that the mental health problems predated the facial pain and they postulate that psychiatric conditions and these facial pains may be mediated by dysfunctional brain dopamine activity. The recent recommendations on rehabilitation of patients with temporomandibular disorders (TMD) also highlights the need to identify patients with mental health problems, termed red and yellow flags and it is suggested that this is done through a combination of questionnaires and clinical interview [26]. Another factor to take into account are the personality differences between doctors and patients as these can significantly affect a consultation [27] and patients anger and frustration with treatment can induce in clinicians emotions of anger/frustration which then hinders the consultation [28].

As in all consultations the history is crucial and clinicians must be prepared to listen to the patients narrative without interruption and with empathy [29]. Langewitz et al. [30] have shown that in a tertiary medical outpatient clinic the mean spontaneous talking time was 92 seconds with 78% of patients having finished their initial statement in two minutes yet physicians re- direct patients’ opening statement after a mean of 23 seconds. In those consultations were patients are allowed to complete their opening statement patient satisfaction is improved and it leads to improved outcomes [31,32]. Cairns et al. [26] have suggest that dentists need additional training when taking histories from patients with TMD so they can identify broader issues which they suggest should include among others, chronicity, functional limitation, discrepancy between findings, overuse of medication, inappropriate behaviours, inappropriate expectations, inappropriate responsiveness to treatments, and risks of self-harm and suicide.

Much as clinicians like to lead their patients through a consultation it is often the uninterrupted opening statement that provides not only diagnostic markers but also details of impact on quality of life. Time is crucial, often restricted, but essential to make a diagnosis and establish a relationship with a patient as good communication is vital. Kenny [15] identified that there is often a struggle in complex pain consultations between the doctor and the patient as both want to function as speakers as both have their beliefs about the condition. This is further compounded by the fact physicians often do not understand their patients’ health beliefs [33]. Good patient doctor relationship will not only improve outcome expectations but will also decrease anxiety [34].

### Classification and diagnosis

The International Headache Society (IHS) is in the process of updating its classification and most conditions covered here are to be found in chapter 13 [35]. Controversy remains about taxonomy and hence lack of international agreement with the result that there are a considerable number of labels being used for what may in fact be the same underlying conditions. An ontological approach would be of immense benefit especially if genotyping is to be done in the future as this would not necessitate any change in nomenclature when underlying mechanisms are identified [36].

**Figure 1:**
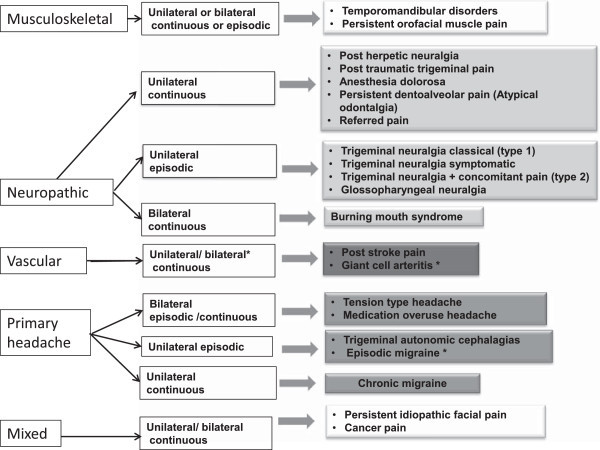
Type and causes of non dental chronic orofacial pain.

When making a diagnosis it is useful to make the distinction whether this is a definite diagnosis, probable or even possible as this allows for a change in diagnosis once more facts come to light. Figure 1 shows a potential schema based on possible causation and presentation of chronic facial pain.

Some chronic facial pain is strictly unilateral and follows clear neurological boundaries whereas others are very widespread in distribution. The majority of pain is continuous with flare ups but there are a few conditions which are very episodic and so it is worth eliciting these factors at the very beginning in order to narrow down the differential diagnosis. Benoliel et al. showed in their clinic population of 328 that chronic orofacial pain could be defined in the same way as chronic daily headache but stress that chronic orofacial pain includes a very heterogeneous group of symptoms [37]. There are a range of conditions that can result not only in pain but associated neuropathy which can be detected either by gross clinical examination or more detailed neurophysiological testing. Conditions that can result in neuropathy include trauma, inflammatory autoimmune disorders e.g. systemic scleroderma, Sjogren’s syndrome, sarcoidosis, multiple sclerosis; rare vascular malformations, neoplasia anywhere along the trigeminal nerve and infections such as leprosy, viral, Lyme disease, syphilis [38]. Table 1 lists the main characteristics of the most common chronic non dental pains.

**Table 1:** The main characteristics of the most common chronic non dental pains and their managment

	**Post traumatic trigeminal neuropathy**	**Buring mouth syndrome**	**Temporomandibular disorders**	**Trigeminal neuralgia**	**Persistent idiopathic facial pain**	**Trigeminal post herpetic neuralgia**
Epidemiology	becoming increasingly common	rare	common	rare	rare	rare
Onset	3-6 months of traumatic event	slow	sometimes starts abruptly	memorable, sudden	slow	slow post herpes zoster
Duration	continuous with minor fluctuations, some have intermittent episodes	continuous	often constant	intermittent seconds to minutes	constant	constant
Periodicity	constant	can vary throughout the day	fluctuations often worse am/evening	refractory periods, many attacks a day periods of complete remission weeks, months	varies, can have periods of no pain	may be excacerbations
Site	distribution of a nerve branch, tooth or tooth bearing area	tongue, lips, palate	masseter, temporalis, around TMJ,ear, retromolar area	V2, V3 most common intraoral and extra oral	non anatomical, gradually gets larger	anatomic distribution, most common ophthalmic branch
Radiation	nil	all parts of the oral mucosa	may radiate to neck	only within trigeminal distribution	can spread over whole face, head, intra oral	little
Character	dull, burning, tingling, pins and needles at times sharp	burning, stinging, sore	aching,heavy, deep, can be sharp	sharp, shooting, lightening, may be a dull ache, burning after pain	dull, nagging, can be sharp	burning,, pins and needles
Severity	moderate to severe	mild to severe	variable moderate to severe	moderate to severe	moderate to severe	moderat to severe
Aggravating factors	touch	sometimes certain food,	prolonged chewing, opening wide, jaw movements	light touch, eating, some attacks are spontaneous	fatigue, stress	light touch,
Associated factors	may be altered sensation, reduced quality of life, history of trauma or dental procedure	altered taste, dry mouth, depression, anxiety, poor quality of life	clenching, bruxism, may have clicking of TMJ, locking, reduced opening, headaches, migraines	very rare autonomic features, fear of pain return, depression, poor quality of life	often other chronic pain, significant life events, vulnerable personalities,	may be altered sensation, skin changes
Examination	allodynia, hypoesthesia	nil, sometimes geographic tongue	palpation of muscles/joint induces same pain, unassisted reduced opening, clicking, intraorally evidence of frictional keratosis in cheeks, attrition of teeth	may trigger attack on touch, very rarely sensory changes	nil	allodynia, hypoaesthesia, hyperaesthesia
Management	drugs for neuropathic pain many benefit from CBT	neuropathic drugs, clonazepam, CBT	education, physiotherapy, psychology, anti- inflammatory drugs	carbamazepine/oxcarbazepine, neurosurgical procedures	CBT, antidepressant drugs	nortryptyline, pregablin, gapabentin, lidocaine patches

CBT cognitive behaviour therapy.

### Dental pain

By far the commonest cause of pain in the lower face is dental i.e. pain related to the teeth and their surrounding structures. Few dental causes are chronic but given its high prevalence it needs to be considered in patients with other chronic pain who report a change in their symptoms which are not expected from the main condition [39,40]. Although some of the dental conditions are easy to diagnose with a careful examination using a good light others will need investigating with local imaging. Dentists are very good at diagnosis of most dental pain and so patients should be referred to them for an assessment. However, beware the dental practitioner who carries out irreversible dental treatment in a patient based on the patient’s history alone with no clinical signs and X-ray validation. Many patients with trigeminal neuralgia have unnecessary teeth extractions and this has been documented by neurosurgeons. In the initial presentation this confusion is understandable as the pain is intermittent and sharp and can seem to the patient to be localised to the teeth. Equally those with neuropathic pain may undergo hours of complex dental treatment and find it does nothing to relieve their pain. The most challenging dental diagnosis is that of the cracked tooth as this can be very difficult to detect and so the symptoms can become chronic. More sophisticated imaging such as cone beam CT may be helpful. Dental pain needs to be treated mechanically together with analgesics and in some cases antibiotics.

### Intraoral non dental pain

Within the mouth there are a variety of other causes of dental pain which are not related to the dental tissues.

Oral mucosal lesions such as recurrent oral ulceration, lichen planus, blistering conditions will cause chronic pain but there are very clear signs which make diagnosis easy. Salivary stones cause intermittent pain of relatively low intensity, pain most frequently occurs at the thought of food and when eating.

In some instances there is a clear history of nerve damage either due to dental procedures or to trauma whereas in other instances it currently remains impossible to determine the mechanism involved in causing the pain. This has led to some confusing nomenclature. Recently an international group of experts proposed the use of the term persistent dentoalveolar pain disorder [36] to encompass persistent pain without local disease (possible other pseudonyms include atypical odontalgia, phantom tooth pain). They propose that the symptoms could then be subdivided into those where there is a clear relationship to some form of trauma and others where the mechanism is unknown. Attempts have been made determine the patients’ experience of this pain and some common themes emerge e.g. difficult to obtain a clear history, complex descriptors, and well localised, deep pain [41]. Terms such as post traumatic trigeminal neuropathy, peripheral painful traumatic trigeminal neuropathy PPTTN could then be used in those instances where there is a clear correlation between trauma and development of pain.

#### *Atypical odontalgia*

In atypical odontalgia the pain is very clearly localised to the dentoalveolar tissues either where teeth are still present or have been lost. There may or may not be a history of dental treatment prior to onset. The pain may move from one area to another. It is a dull throbbing continuous pain which can at times be sharp. It is often light touch provoked. Baad-Hansen postulates it is most likely to be a neuropathic pain and has different features from TMD [42,43]. Thus attempts have been made to characterise these patients but there is little evidence that pharmacological treatment along the lines of a neuropathic pain provide relief [43,44] but it probably is important to avoid more surgical interventions which can result in increased sensitisation.

#### Post traumatic trigeminal neuropathy

In some instances there is a clear history of nerve injury which can range from a dental extraction, root canal filling [45], local anaesthetic [46], implants [47], facial trauma [48]. Nerve injury, of varying degree, can be assumed to have been the cause and these conditions could be called a post traumatic trigeminal neuropathy or peripheral painful traumatic trigeminal neuropathy PPTTN as recently proposed by Benoliel et al. [48]. Benoliel et al. compared 91 PPTN with 54 classical trigeminal neuralgia (TN) patients and showed that the temporal features of PPTTN were very varied with only 50% having continuous pain whereas others reported daily pain but which lasted less than 4 hours, some even had very short attacks that were similar to TN like pain. Pain is often described as burning, stabbing [43,48] but patients with definite nerve injury such as lingual and inferior alveolar nerve describe a feeling of pins and needles, fizzing and swollen sensations [47]. Pain in some circumstances is evoked in others it is spontaneous. Some sensory changes can often be detected on clinical testing and somatosensory testing will often show evidence of hypoaesthesia or allodynia, most imaging is negative. Meyer et al. have proposed a careful evaluation of peripheral nerve injuries that is both subjective (history, questionnaires and examination) and objective which includes quantitative sensory testing and imaging [49]. Renton et al. have shown how quality of life can be affected by trigeminal nerve injuries and the negative effects are more pronounced in patients with inferior alveolar nerve injury as opposed to lingual nerve injury [47]. Not all traumatic injuries result in pain some only present with sensory changes [47]. Although surgical repair is possible for inferior alveolar nerve most patients need to be managed according to guidelines for neuropathic pain.

#### Burning mouth syndrome glossodynia, stomatodynia

**Figure 2:**
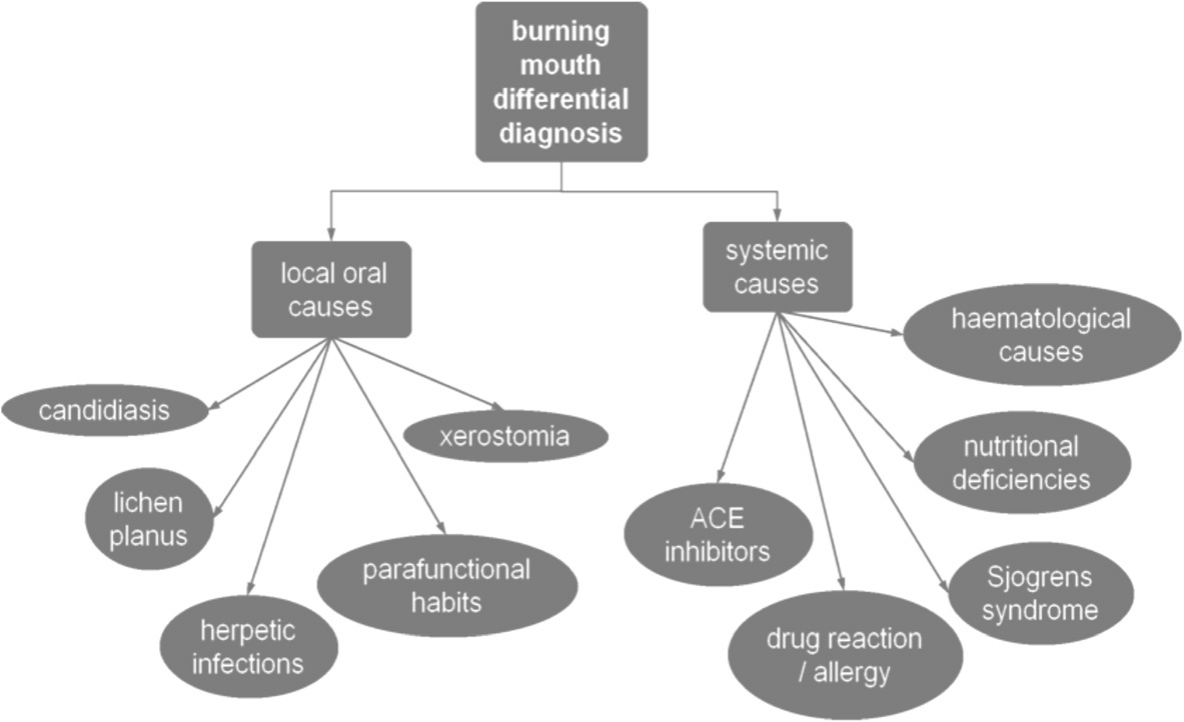
Causes of burning mouth.

This strictly intraoral condition presents as a burning, discomfort of the oral mucosa especially the tongue for which local or systemic causes cannot be found. It is unusual in that it is especially common in post-menopausal women who have been found to have a high level of anxiety and depression. The aetiology remains unknown and a variety of hypothesis have been put forward [50]. Neurophysiological testing and biopsies of the tongue have indicated that there are peripheral nerve changes with abnormal appreciation of temperature but central changes have also been noted on fMRI testing [51]. Not only do the patients report abnormal sensations but there are often other symptoms such as altered taste and disturbed salivary production [52]. The symptoms can be continuous but the intensity does vary throughout the day and some patients find eating mild food helpful. On examination the oral mucosa is normal although it is not unusual to see signs of a geographic tongue (erythema migrans) or fissured tongue and several patients have a habit of thrusting the tongue against their front teeth. Investigations are needed to exclude other causes of burning as indicated in Figure 2.

Management begins with acknowledging the symptoms as real and reassuring the patient that it is a recognised albeit rare condition. There have been a number of RCTs performed and a Cochrane systematic review [53]. Although a small study, not replicated again, cognitive behaviour therapy has been shown to be effective and it is the mainstay management in our facial pain unit. Clonazepam as a topical and systemic agent has been evaluated with mixed outcomes [54,55] but the significant side effect of drowsiness and the potential for addiction need to be taken into account. There are several RCTs on the use of alphalipoic acid, an antioxidant, but the outcomes are conflicting [53]. Given the evidence that this is potentially a neuropathic pain then drugs for neuropathic pain e.g. gabapentin, pregablin, tricyclic antidepressants can be tried. There is little data on prognosis. Sardella et al. in a survey of 53 patients showed that 3% had complete spontaneous resolution and less than 30% had a moderate improvement [56].

### Extraoral pain with or without intra oral pain

These conditions often present with pain in both locations but equally can occur only extra-orally.

#### Temporomandibular disorders TMD

By far the commonest cause of non dental pain is a musculoskeletal pain related to the masticatory apparatus and it is principally an extra oral pain but pain is often felt in the retromolar area. TMD encompasses a variety of different conditions and most of the research in this area has been carried out by dentists with a specific interest in facial pain. The Research Diagnostic Criteria (RDC) for TMD where published in 1992 and have been used as a basis for research internationally [57]. The criteria include psychological distress and quality of life assessments which large studies in the US currently being done show are of great importance in prognosis [58,59]. Recently the RDC has been updated to make it more clinically relevant [60,61]. In the new proposals the commonest condition will be called myalgia and the criteria are: pain in the muscle affected by jaw movement and palpation of the masseter or temporalis induces the same pain. If the pain radiates to other local structures then it will be termed myofascial pain with referral. Disorders of the disc within the joint lead to clicking and crepitus and if the disc does not reduce then it will lead to limitation of opening and possible locking. On their own discs do not cause pain but they are often found in association with muscle pain. Degenerative disorders, joint dislocations as often found in hyper or hypomobility conditions do not cause significant pain. It is increasingly being recognised that TMD pain is related to other conditions such as fibromyalgia, back pain, migraine. It is also proposed that some headaches are due to TMD in that jaw movement induces the same headache [62]. Patients with TMDs do not cope as well with stress as general population [63] and oral health related quality of life is negatively affected by this condition becoming more pronounced in those with more symptoms [64].

A wide range of therapies are used but overall self-management through education, physiotherapy and with some cognitive behaviour therapy needs to be encouraged. Cairns et al. stress that patients with co-morbidities should be seen by specialists with training in pain management [26]. Therapies range from soft diet, splint, and physiotherapy, drugs, psychological and surgical. Many of these have been evaluated in RCTs and there are several systematic reviews [65-67] but the quality of many of the trials is poor. A variety of surgical procedures including arthocentesis and arthroscopy can be used but they should only be used if there are functional signs [68].

#### *Trigeminal neuralgia*

Trigeminal neuralgia (TN) defined by The International Association for the Study of Pain (IASP) “as a sudden usually unilateral severe brief stabbing recurrent episodes of pain in the distribution of one or more branches of the trigeminal nerve.” [69] and has been shown to have a significant impact on quality of life [70]. TN presents most commonly in the lower two branches of the trigeminal nerve. Very often it presents intraorally with triggers around the teeth [71] and hence many patients will undergo irreversible dental treatment unnecessarily [72].

It is easy to misdiagnosis TN as TMD especially if the TMD is unilateral as it is a far more common condition [73] whereas GPs tended to over diagnosis this condition [9]. Although it is often considered that TN is easy to diagnosis there is an increasing understanding that TN has a varied presentation and that some patients report considerable amount of less intense burning or dull pain after the main sharp attack of pain which can be present for more than 50% of the time. These have variously been labelled as type 2 [74] or TN with concomitant pain [75]. Neurosurgeons have started to report that outcomes are different in these groups [76]. Of the trigeminal autonomic cephalalgias SUNA (short unilateral neuralgiform pain with autonomic symptoms) needs to be considered as a potential differential as it can present in the lower face [77].

Symptomatic causes of TN need to be excluded e.g. tumours, often benign, multiple sclerosis and A-V malformations. Imaging is now considered to be part of the routine workup and some centres will use qualitative sensory testing [78,79].

Management is first medical with carbamazepine or oxcarbazepine with second line drugs being lamotrigine and baclofen [80,81]. If patients develop significant side effects or have poor pain control then surgery needs to be considered [78,79]. There are very few RCTs of surgical treatments [82] and evidence is based mainly on cohort data [78,79]. Microvascular decompression is the only procedure that is non-destructive and gives the longest pain free interval 70% pain free at 10 years [83]. For patients not suitable for this procedure ablative procedures include radiofrequency thermocoagulation glycerol rhizotomy, balloon compression or Gamma knife and these give 50% pain relief for four years but patients risk sensory changes which impact on quality of life [78,79]. Patient support groups are invaluable in providing further information and helping patients make decisions about treatments [84].

#### *Glossopharyngeal neuralgia*

This is a very rare condition and has the same features as TN except for location and the two conditions have also been reported to co-exist. Pain can be felt deep within the ear but more commonly in the back of the tongue and throat. Talking and swallowing are particular trigger factors. Medical management is as for TN and in poorly controlled patients microvascular decompression is the surgery of choice [85].

#### Anaesthesia Dolorosa/post traumatic trigeminal neuropathy

Anaesthesia dolorosa is a term used by the neurosurgeons to denote pain after surgical damage to the trigeminal nerve most commonly at the level of the Gasserian Ganglion which occurs after ablative procedures for TN [74]. When the cause is due to other trauma e.g. fractures the term post traumatic trigeminal neuropathy is used. Both of these conditions develop within 3–6 months of the traumatic incident. The distribution is varied depending on the extent of the trauma but often in trigeminal neuralgia patients it extends to all three divisions of the trigeminal nerve. Patients report hyperalgesia, allodynia, hypoaesthesia and hypoalgesia very often described as “ants crawling over the face”. There are often associated extensive psychological factors e.g. anger, depression, present and management is extremely difficult. In trauma patients the symptoms are often less severe and more localised. Patients report a poor response to drugs used for neuropathic pain and addition of cognitive behaviour therapy may be of value.

#### Persistent Idiopathic facial pain/Atypical facial pain

This condition has often been used as a bucket term to include all other facial pain that does not fit into other criteria as these patients do not have any sensory or physical signs. A recent small study on this group of patients using voxel-based morphotometry indicates that they do have similar changes to other chronic pain patients in parts of the brain associated with pain [86]. Epidemiological studies and psychiatric assessments suggest that psychological factors play a role in this condition [1,12] and that many of these patients are likely to have other chronic pain elsewhere [7]. This is generally a continuous pain which does not follow a neurological distribution. The pain often gradually becomes more diffuse and involves a larger area of the head and neck. Although often described as nagging and dull it can have sharp exacerbations [87]. All investigations are normal although Forssell et al. have shown that some patients labelled with this condition may on neurophysiological testing have abnormalities similar to trigeminal neuropathic pain [88]. Management is difficult and all types of approaches have been used - pharmacotherapy and behavioural therapy [89,90]. A multi-disciplinary approach using a combination of antidepressants drugs and cognitive behaviour therapy may be the best form of management [91].

#### Giant cell arteritis

Although principally occurring in the temple region jaw movement can induce more widespread pain and the tongue can be affected to such an extent that it appears cyanosed. The criteria are well established and any patient over 50 years should have an ESR and C reactive protein test. Temporal artery biopsy has been used as the gold standard but it is now suggested that ultrasonography and MRI scanning may be equally diagnostic [92]. Systematic steroids need to be commenced promptly to prevent blindness and other systemic effects [93].

#### Facial migraine/Neurovascular orofacial pain

There are several papers that have documented migraine like features of the lower face or as Benoliel et al. have called them neurovascular orofacial pain [94,95]. Yoon et al. [96] assessed migraine sufferers in the population and they established that this condition was rare but found that 9% of patients with migraine can have symptoms in the lower face i.e. V2 and 3 distribution but it was very rare to have symptoms only in the lower face. Benoliel et al. in their series of 328 patients diagnosed this condition in 23 patients. The pain was episodic or chronic, high severity, located in the lower half of the facial, (bilateral or unilateral), seven reported nausea and photophobia, eight autonomic features. It was not clear whether the headache was disabling and whether there was associated aura. Response to anti-migraine therapy including triptans was not provided. Oberman in their series of 7 showed all responded to triptans and three to prophylactic measures [94].

## Conclusions

There are many different causes of orofacial pain and given the wide range of aetiologies management is also varied so that diagnosis is important in order to use the correct care pathway [40]. It is crucial to take a multidimensional approach to these patients and they are best managed in centres which have multi-disciplinary teams including pain specialists, oral surgeons, liaison psychiatrists, headache neurologists, neurosurgeons, clinical psychologists, physiotherapists and radiologists [4].

## Competing interests

The author declare that she has no competing interests.
